# A Case of Acute Pancreatitis after Pancreatectomy in Grade C Leading to Walled-Off Necrosis Successfully Treated with Necrosectomy by Retroperitoneal Approach

**DOI:** 10.70352/scrj.cr.24-0002

**Published:** 2025-02-01

**Authors:** Makoto Shinohara, Masakazu Hashimoto, Ryo Nagao, Michinori Hamaoka, Masashi Miguchi, Nobuaki Fujikuni, Satoshi Ikeda, Yasuhiro Matsugu, Hideki Nakahara

**Affiliations:** Department of Gastroenterological Surgery, Hiroshima Prefectural Hospital, Hiroshima, Hiroshima, Japan

**Keywords:** post-pancreatectomy acute pancreatitis, walled-off necrosis, necrosectomy

## Abstract

**INTRODUCTION:**

Grade B or C post-pancreatectomy acute pancreatitis (PPAP) is associated with a higher incidence of postoperative complications and mortality. The reason for this is the activation of proteolytic processes that can lead to pancreatic destruction and the activation of systemic reactions that can have adverse consequences such as systemic inflammatory response syndrome, sepsis, and death. We report a case of a patient with Grade C PPAP with walled-off necrosis (WON) who was successfully treated with necrosectomy using a step-up approach.

**CASE PRESENTATION:**

A 73-year-old man was referred to our hospital with elevated biliary enzymes. Results of blood tests, computed tomography (CT), and magnetic resonance imaging led to the diagnosis of distal bile duct cancer. He underwent a pyloric ring-sparing pancreaticoduodenectomy with lymph node dissection. Postoperative P-AMY (pancreatic amylase) was high at 1766 U/L, and contrast-enhanced CT showed increased density of peripancreatic fatty tissue and fluid accumulation on the pancreatic resection surface, leading to the diagnosis of postoperative pancreatitis and pancreatic fistula. On postoperative day (POD) 9, continuous washing with saline solution was started through the drain at the pancreatic anastomosis. Contrast-enhanced CT showed increased fluid retention in the pancreatic body tail. On POD 43, endoscopic ultrasonography drainage was performed for pancreatic necrosis encapsulated in the retroperitoneum; however, the patient self-extracted the drainage tube. On POD 50, CT-guided drainage was performed for a retroperitoneal subcapsular abscess. On POD 69, the patient underwent necrotomy with guided retroperitoneal drainage, a drain was inserted, and continuous flushing was performed. On POD 76, fecal discharge was observed from the drain, and drainage and enterography were performed; a fistula with the colon was confirmed, and an ileal bifurcation colostomy was performed on the same day. On PODs 83, 85, and 100, endoscopic necrotomy was performed through a retroperitoneal incision wound because a contrast-enhanced CT showed a residual abscess on the gastric dorsum. The patient’s general condition improved, and his inflammatory response also improved. On POD 139, the patient was transferred for rehabilitation.

**CONCLUSION:**

We describe a case of successful postoperative nutritional management and necrosectomy for Grade C PPAP leading to WON.

## Abbreviations

PPAP
: post-pancreatectomy acute pancreatitis
TEN
: total enteral nutrition
WON
: walled-off necrosis
ERCP
: endoscopic retrograde cholangiopancreatography
DGE
: delayed gastric emptying
POPF
: postoperative pancreatic fistula
PPH
: post-pancreatectomy hemorrhage
PPN
: peripheral parenteral nutrition
TPN
: total parenteral nutrition
POD
: postoperative day
EUS
: endoscopic ultrasonography

## INTRODUCTION

Post-pancreatectomy acute pancreatitis (PPAP) is defined as “a condition in which the remainder of the pancreas becomes acutely inflamed after pancreatectomy, with early onset within 3 days after surgery.”^1)^ This pathophysiologic process exhibits varying degrees of severity and can lead to local and systemic complications. A significant increase in complications of Clavien-Dindo classification IIIb or higher has been reported in patients on PPAP, as well as peritonitis, intra-abdominal infection, ascites accumulation, puncture and drainage procedures, and mortality.^2)^

In this case, a necrosectomy by retroperitoneal approach was performed to save a patient with PPAP with walled-off necrosis (WON) after pancreaticoduodenectomy.

## CASE PRESENTATION

**Fig. 1:**
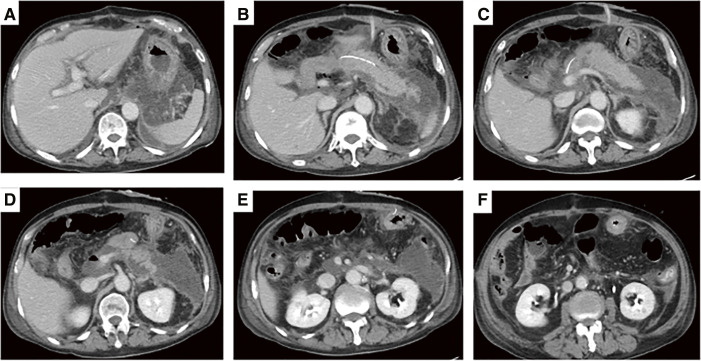
On POD 9, contrast-enhanced CT at the onset of postoperative pancreatitis. (**A–F**) Pancreatic swelling and fluid accumulation around the pancreatico-jejunal anastomosis and in the dorsal gastric region. POD, postoperative day; CT, computed tomography

**Fig. 2:**
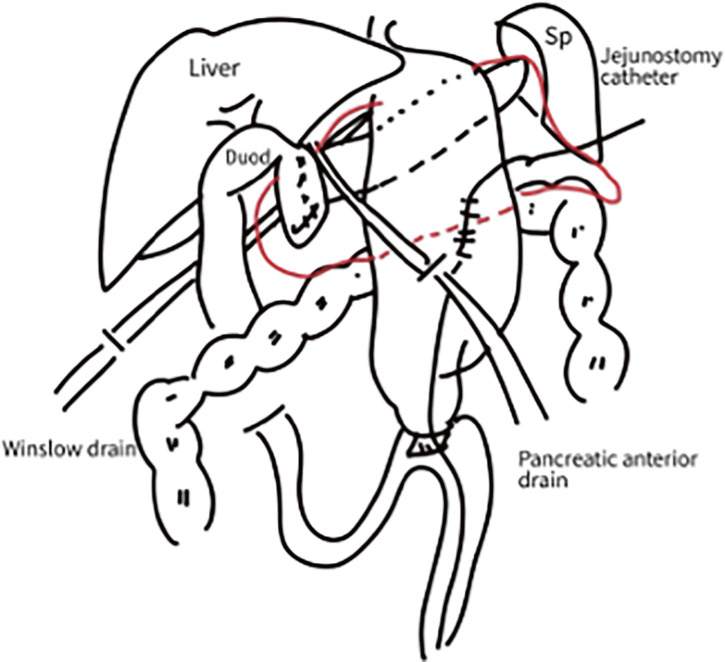
Pancreaticoduodenectomy was performed. Reconstruction was performed according to the Child method. A jejunostomy catheter was placed intraoperatively, and the operation was completed with drains placed in the Winslow and anterior pancreas. The area surrounded by a red line shows fluid retention. Duod, duodenum; Sp, spleen

**Fig. 3:**
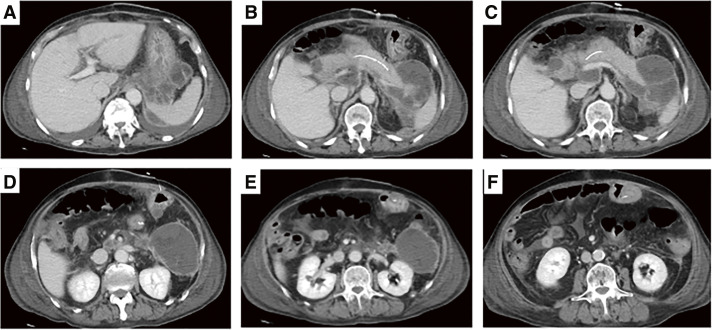
(**A–F**) On POD 39, fluid retention around the pancreatic jejunal anastomosis decreased. Diffuse peripancreatic fluid accumulation showed necrosis and encapsulation. POD, postoperative day

**Fig. 4:**
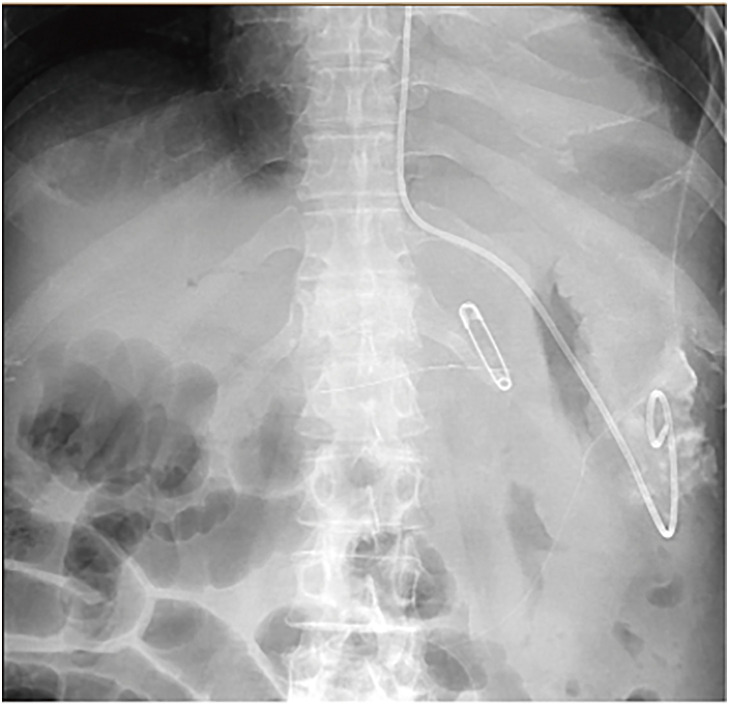
On POD 43, a drainage procedure was performed under EUS for encapsulated pancreatic necrosis on the dorsal gastric side. POD, postoperative day; EUS, endoscopic ultrasonography

**Fig. 5:**
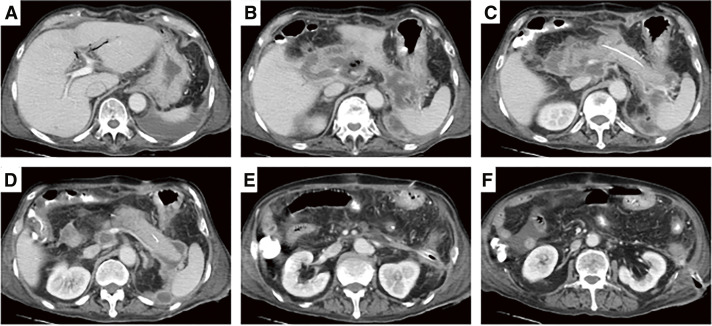
(**A–F**) On POD 82, contrast-enhanced CT revealed a residual abscess posterior to the stomach. POD, postoperative day; CT, computed tomography

**Fig. 6:**
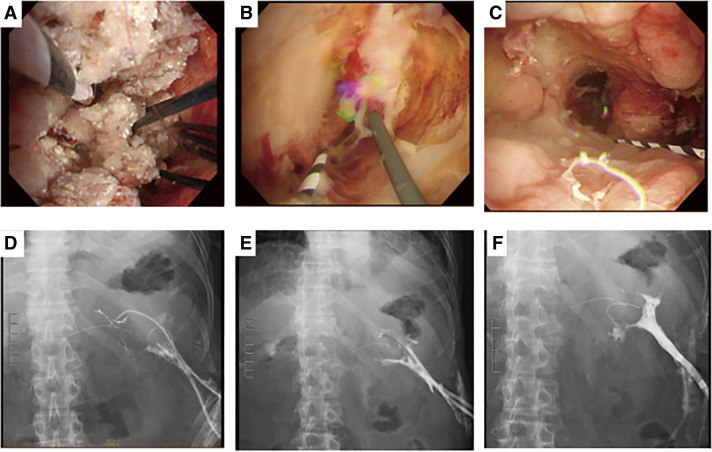
Endoscopic necrosectomy was performed through a retroperitoneal incision wound a total of three times. (**A, D**) First time (POD 83), (**B, E**) second time (POD 85), and (**C, F**) third time (POD 100). The third time was cleaner than the first and second times, and good granulation was confirmed. POD, postoperative day

**Fig. 7:**
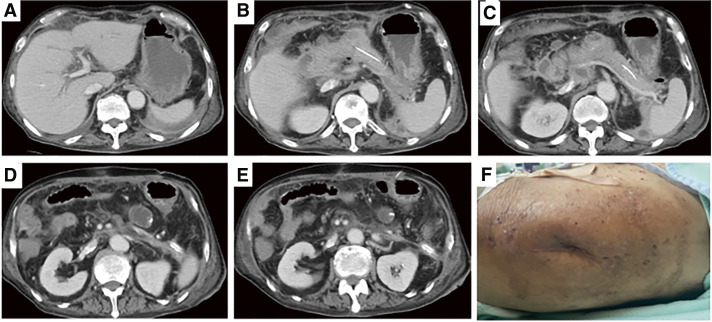
CT images and wound findings on POD day 115. (**A–E**) Encapsulated necrotic tissue has shrunk and fluid retention has improved. (**F**) Complete closure of the wound has been achieved. CT, computed tomography; POD, postoperative day

**Fig. 8:**
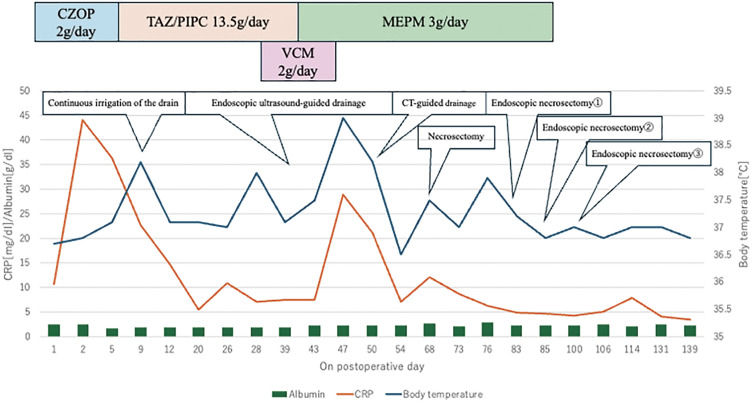
Time course of inflammatory markers and therapeutic interventions. The patient’s serum CRP and albumin levels (left axis) and body temperature (right axis) are shown over a period of approximately 139 days, post-surgery. Initial treatment with CZOP (2 g/day) is marked by a blue box, followed by TAZ/PIPC (13.5 g/day, orange box). VCM (2 g/day, pink box) was used in addition to TAZ/PIPC. We used TAZ/PIPC replaced with MEPM (3 g/day, green box). CZOP, cefozopran hydrochloride; TAZ/PIPC, tazobactam/piperacillin; VCM, Vancomycin; MEPM, meropenem; CT, computed tomography; CRP, C-reactive protein

A 73-year-old man was referred to our hospital because of elevated serum alkaline phosphatase (ALP) and gamma-glutamyl transpeptidase (γ-GTP) levels. He had a history of atrial fibrillation and cerebral infarction, but no family history, abdominal surgery, or allergies were noted. He had a history of smoking but no history of heavy alcohol consumption. The patient’s Performance Status was 1. Laboratory examination results showed elevated serum aspartate aminotransferase (137 U/L; reference: 13–30 U/L), alanine aminotransferase (139 U/L; reference: 17–23 U/L), ALP 526 U/L, γ-GTP 1071 U/L. Total bilirubin (T-Bil) was not elevated. The patient was negative for hepatitis virus and had elevated serum tumor markers (carcinoembryonic antigen [CEA] and carbohydrate antigen 19-9 [CA19-9]), and IgG4 levels were within the normal limits. Contrast-enhanced computed tomography (CT) showed wall thickening with thickened intrapancreatic bile ducts, upper common bile duct, and intrahepatic bile duct dilatation. The liver parenchyma showed speckled early staining, suggesting cholangitis-induced changes. Magnetic resonance imaging showed a signal defect in the bile duct, endoscopic retrograde cholangiopancreatography (ERCP) showed a 2 cm stenosis in the lower bile duct, and intraductal ultrasonography showed a circumferential wall thickening at the site of stenosis. Biliary cytology, brushing cytology, and biopsy were performed, but no malignant findings were proven in all specimens. Since distal cholangiocarcinoma was strongly suspected on endoscopic and radiographic findings, surgical treatment was embarked on with the patient’s full consent. A pylorus-preserving pancreaticoduodenectomy with regional lymph node dissection was performed. Intraoperatively, pancreatic duct cannulation was performed; reconstruction was carried out using the Child’s method. Pancreatic-jejunal anastomosis was completed using the Blumgart technique. 19Fr Blake drains (Ethicon, Somerville, New Jersey, USA) were placed in the foramen of Winslow and anterior to the pancreatic anastomosis, completing the procedure. Histopathological findings revealed proliferating tumor tissue in the distal bile duct showing a 55 × 25 mm foci-like structure. The tumor primarily manifested as a poorly differentiated ductal adenocarcinoma. Although the tumor invaded the peri-bile duct tissue into the sub-serosal tissue, there was no invasion into the pancreatic tissue. Extension into the duodenal papillary region was noted. Lymphatic and venous invasion was noted. However, no lymph node metastasis was observed. The pathological diagnosis, according to the TNM staging system of the Union for International Cancer Control 8th edition, was Stage IIA (T2 N0 M0) distal bile duct cancer. On postoperative day (POD) 2, vomiting and abdominal distention were observed, and white blood count (WBC; 15700/μL), C-reactive protein (CRP; 44 mg/dL), serum pancreatic amylase (P-AMY; 1766 U/L), trypsin (36000 ng/mL), lipase (1462 U/L), and drain AMY (6875 U/L) were elevated. Nafamostat mesilate and ulinastatin were administered continuously up to POD 5. In addition, the antibiotic cefozopran hydrochloride (2 g/day) was used continuously from the day of surgery. On POD 3, the drain AMY content was greater than three times the serum AMY; a pancreatic fistula was diagnosed. On POD 9, contrast-enhanced CT showed increased peripancreatic fatty tissue density, fluid accumulation in the pancreatic resection surface to the superior mesenteric vein and around the portal vein, and a diagnosis of postoperative pancreatitis was made (**Figs. 1** and **2**). On POD 9, the pancreatic drain in front of the anastomosis that had been placed intraoperatively was replaced with a 14Fr Nelaton catheter (Terumo Corporation, Tokyo, Japan). Continuous irrigation with normal saline was initiated through the catheter to prevent post-pancreatectomy hemorrhage (PPH) due to poor drainage. On POD 25, the 14Fr Nelaton catheter on the anterior surface of the pancreas was replaced with a 10Fr one; irrigation was continued. On POD 39, contrast-enhanced CT showed that the intra-abdominal cavity due to pancreatic juice disappeared, but there was an increase in fluid in the pancreatic body tail (**Fig. 3**). On POD 43, an endoscopic ultrasonography (EUS) drainage procedure was performed for encapsulated pancreatic necrosis in the retroperitoneum (**Fig. 4**). Endostomy was planned, but on POD 46, the patient developed fever after self-removal of the drainage tube. *Enterobacter cloacae* was identified using a culture obtained from the abscess drainage during this procedure. On POD 50, the patient underwent CT-guided drainage via the retroperitoneum for encapsulated pancreatic necrosis of the retroperitoneum. The same bacteria as those detected in EUS drainage were detected. Based on the detection of bacteria from the abscess drainage culture, persistent inflammatory responses with WBC (13000/μL) and CRP (12.15 mg/dL), as well as the presence of fever, the patient underwent necrosectomy with guided retroperitoneal drainage on POD 69. A drain was placed in the abscess cavity located on the dorsal side of the stomach; continuous irrigation was performed. On POD 76, fecal discharge was observed from the drain, and drainage and enterography were performed to confirm a fistula with the colon, and an ileal bi-pore colostomy was performed on the same day. On POD 82, contrast-enhanced CT revealed a residual abscess posterior to the stomach (**Fig. 5**), with persistently elevated inflammatory markers. Therefore, endoscopic necrosectomy via retroperitoneal incision was performed on PODs 83, 85, and 100 (**Fig. 6**). The patient’s general condition improved, and the inflammatory response also improved (**Fig. 7**). For postoperative nutritional management, peripheral parenteral nutrition (PPN) was administered intravenously from PODs 1 to 11, and total parenteral nutrition (TPN) was started and administered from POD 12. Enteral nutrition was administered via enterostomy starting from POD6. However, after 3 endoscopic necrosectomies, the inflammatory response decreased, and the fluid retention improved on the CT scan. The patient was transferred to the hospital for rehabilitation on POD 139 (**Fig. 8**).

## DISCUSSION

This is a case of WON triggered by PPAP, which was successfully treated by necrosectomy via retroperitoneal approach and saved the patient’s life.

The International Study Group for Pancreatic Surgery (ISGPS) defines PPAP as an acute inflammatory condition of the pancreatic remnant occurring in the setting of a partial pancreatic resection and initiated early in the perioperative period within the first 3 PODs.^1)^ To diagnose PPAP, a sustained increase in serum amylase activity above the institutional upper limit within the first 48 hours postoperatively is required. PPAP must also be confirmed by cross-sectional imaging and be clinically relevant. PPAP severity is classified into Grade B and C. Grade B PPAP involves the following: (1) a sustained increase in serum amylase activity within the first 48 hours postoperatively, (2) a clinically relevant downturn in the patient’s condition, and (3) radiologic abnormalities consistent with PPAP, such as inflammatory enlargement of the pancreatic remnant, changes in the peripancreatic fat, fluid collections, or necrosis. If Grade B PPAP results in persistent organ failure or necessitates surgery, it is elevated to Grade C.^1)^

Factors reported to influence the development of PPAP include the female, soft texture of pancreatic tissue, pancreatic ducts less than 3 mm, additional resection of the pancreatic stump margin, and a different pathology type than Pancreatic Invasive Ductal Adenocarcinoma or Chronic Pancreatitis.^2–4)^ Factors affecting the development of PPAP, in this case, were the soft texture of pancreatic tissue due to distal cholangiocarcinoma, 2.5 mm pancreatic duct, which is a different disease type from invasive mastoid carcinoma and chronic pancreatitis. The soft pancreas and duct were less than 3 mm, which is assumed to be the cause.

There is no established treatment for PPAP, but experience has shown that it is commonly treated similarly to acute pancreatitis, with reduced food intake and the use of somatostatin analogs and antibiotics.^3)^ It has also been reported that the anti-inflammatory effect of hydrocortisone as prophylaxis for PPAP may reduce postoperative pancreatic inflammation, thereby lowering complications after pancreaticoduodenectomy.^5)^ Clavien-Dindo ≥ IIIb complications were significantly increased in patients who developed PPAP, as were clinically relevant pancreatic fistulas, intra-abdominal infections, ascites effusions, punctures, drainage procedures, and mortality.^2)^ With regard to short-term outcomes, a strong correlation between the development of PPAP and more severe clinical outcomes has already been reported. Specifically, the development of grade B and C PPAP was associated with higher rates of serious postoperative complications, delayed gastric emptying (DGE), postoperative pancreatic fistula (POPF), PPH, and re-operation.^6)^ The patient underwent pylorus-preserving pancreaticoduodenectomy (PPPD) for distal bile duct cancer. Reconstruction was performed according to Child’s procedure. The patient had persistent hyperamylasaemia for more than 48 hours postoperatively; contrast-enhanced CT showed increased peripancreatic adipose tissue density and peripancreatic effusion. PPAP diagnosis was made, and treatment with fasting, antibiotics, and Nafamostat Mesilate for pancreatitis was started. However, the postoperative course showed worsening respiratory status and coagulation dysfunction due to cardiac failure, and a diagnosis of PPAP Grade C was made.

According to the 2012 revision of the Atlanta classification of acute pancreatitis,^7)^ peripancreatic collections are classified according to the time since the onset of acute pancreatitis. Up to 4 weeks after onset, the collection is a mixed reservoir of fluid and necrotic material in varying proportions and is termed an acute necrotic collection (ANC). After 4 weeks, collections usually have definite limits and mature walls and are called pseudocysts or WON, depending on the absence or presence of solid necrotic material.^8)^

The major cause of death is the infection of the necrotic tissue, which is associated with a poor prognosis: mortality is approximately 15% in patients with necrotizing pancreatitis (NP) and up to 30%–39% in those with infected necrosis. PPAP complicated by POPF significantly increases the incidence of mortality, Clavien-Dindo ≥ IIIb complications, PPH, DGE, intra-abdominal infection, ascites effusion, percutaneous drainage, and unplanned secondary surgery. To the best of our knowledge, this is the first case report of documented life-saving necrosectomy in patients with NP due to POPF complicated by PPAP. Rudis et al.^9)^ found that grade C pancreatic fistula (PF) complicated with PPAP was observed in 4 out of 160 patients, and none of these patients survived. The treatment of NP is open drainage and necrosectomy,^10)^ but this invasive approach is associated with a high complication and mortality rate. To reduce this risk, current guidelines for NP recommend a surgical step-up approach, which has been shown to reduce the risk of serious complications and death from the open necrosectomy approach from 69% to 40%.^11,12)^ Endoscopic tubal drainage, percutaneous catheter drainage, endoscopic or laparoscopic necrotomy, and trans-retroperitoneal or open necrotomy have been proposed as approaches. These techniques can be performed in a stepwise approach that progressively intensifies according to therapeutic effect from minimally invasive to invasive approaches.^13)^ The treatment process, in this case, involved continuous drain flushing after the onset of PPAP; after 4 weeks, transendoscopic drainage was performed on POD 43 when the WON was complete. Unfortunately, the drainage tube was self-removed, and percutaneous drainage was performed on POD 50. CT showed a shrinkage of the abscess, but drainage decreased, and the inflammatory response increased again, so an open necrosectomy was performed on POD 69 via a retroperitoneal approach. The patient underwent a total of three endoscopic necrosectomies through retroperitoneal incisions. The inflammatory response decreased, and the patient was transferred to a rehabilitation center on POD 139.

One notable aspect of this case is that one of the reasons the patient’s life was saved was perioperative nutritional management. Postoperative management with enhanced recovery after surgery (ERAS) by Matsugu et al.^14)^ uses a transgastric catheter-jejunal anastomosis in pancreatic resection cases in which patients with postoperative complications such as PPAP, PF, or DGE cannot receive early oral nutrition or may not meet nutritional requirements if they do. Although enterocutaneous fistula is not recommended in all patients, our institution has created enterocutaneous fistulas in patients who are (1) over 75 years old, (2) at high risk for pancreatic fistula (Pancreatic Fistula Risk Score >7 points), and (3) on dialysis or have Parkinson’s disease. A number of studies have demonstrated that early initiation of enteral nutrition in patients with severe pancreatitis is associated with significantly improved outcomes.^15)^ Patients receiving total enteral nutrition (TEN) have been reported to have a significantly lower incidence of pancreatic infection complications, multi-organ failure, and death compared to patients receiving TPN.^16,17)^ In the present case, the patient was also unable to eat postoperatively; however, by administering enteral nutrition via jejunostomy in addition to intravenous nutrition, his nutritional requirements were met, although not fully met. This approach was considered effective for the patient’s recovery.

## CONCLUSION

In our case, we were able to save the life of the patient with Grade C PPAP with a low survival rate of WON by performing a necrosectomy in a step-up approach. Necrosectomy approach and nutritional management are important.

## ACKNOWLEDGMENTS

This study was not supported by any grant.

## DECLARATIONS

### Funding

The authors declare that they received no funding support for this study.

### Authors’ contributions

MS and MH wrote the manuscript. MS, MH, and RN were involved in the patient’s clinical management.

YM and HN revised the manuscript.

All the authors have read and approved the final version of the manuscript.

### Availability of data and materials

Not applicable.

### Ethics approval and consent to participate

Not applicable.

### Consent for publication

Informed consent was obtained from the patient and his family for the publication of this report.

### Competing interests

The authors declare that they have no competing interests.
